# Carbolic Acid

**Published:** 1885-09

**Authors:** 


### Carbolic Acid.

In many parts of the country, every farmer has his smoke-house, where he hangs up his bacon and hams in the fall of the year, kindles a chip-fire, so that it will not burn but cause a dense smoke. This is continued many days as a means of preventing the meat from decaying. The principle in the smoke which thus prevents decay, is carbolic acid, discovered as a separate substance in 1834. It is obtained from the "soot" in our chimneys, and is really a refined creosote. The more its properties were studied by chemists, the greater appeared to be its value, until at present it is an important article of commerce, and is required in such large quantities that extensive manufactories have been built. It was first made from wood-tar, but is more cheaply obtained from coal-oil by heating it to a high point, a distilling process, and the chemists give it to us in the shape of colorless crystals, and also in a liquid form. Its value consists in its being the best thing known to prevent and arrest decay, and is hence called a "disinfectant," removing the bad odors from all decaying substances with a promptitude, a certainty, and a completeness peculiar to itself. It is an acrid poison if taken into the stomach, and, if kept about the house, should be so marked in large, plain Roman letters. It has so many domestic uses that the time may come when it will be kept in many households. Its employment is not advised, except by medical direction, unless it be to dispel bad odors in cellars, sinks, sick-rooms, gutters, dirt-heaps and wherever there is any vegetable decay; and when it is remembered that it is vegetable decay which causes diarrhœas, dysenteries, fevers, and cholera in whole communities, the judicious use of carbolic acid becomes an important safeguard, especially when epidemic diseases prevail; first scrupulously cleaning out every hole and corner about the premises, and then sprinkling the carbolic mixture freely around; a pound of the acid dissolved in five gallons of hot water. But cleanliness is better than carbolic acid.

WATER is purified by the process recommended by Profs. Almen and Hausemann: To each quart of suspected water add about fifty minims of a three per cent. solution of muriate of iron, and afterwards lime water in inverse proportion to the hardness of the water—say one and a half ounces for a quart of ordinarily hard water. Now filter, and the filtrate will be almost pure, as seventy to eighty per cent. of organic matter will be thus removed.—*Rundschau Leitmeritz—National Druggist.*

